# Expression and Roles of Free Radicals and Reactive Oxygen Species in Hearing Loss

**DOI:** 10.3390/antiox14121397

**Published:** 2025-11-24

**Authors:** Jae Min Lee, Yoo Jin Cha, Yeon Ju Oh, Hye Ok Kim, Sung Soo Kim, Youn-Jung Kim, Dong Keon Yon, Seung Geun Yeo

**Affiliations:** 1 Department of Otorhinolaryngology Head & Neck Surgery, Kyung Hee University School of Medicine, Kyung Hee University Medical Center, Seoul 02447, Republic of Korea; jmlee3042@khu.ac.kr; 2 Department of Medicine, College of Medicine, Kyung Hee University Medical Center, Seoul 02447, Republic of Korea; swj09161@khu.ac.kr (Y.J.C.); 5duswn1203@khu.ac.kr (Y.J.O.); hyeokkim@khu.ac.kr (H.O.K.); 3 Clinical Research Institute, Kyung Hee University Medical Center, Seoul 02447, Republic of Korea; 4 Medical Research Center for Bioreaction to Reactive Oxygen Species and Biomedical Science Institute, Core Research Institute (CRI), Kyung Hee University, Seoul 02447, Republic of Korea; sgskim@khu.ac.kr; 5 Department of Basic Nursing Science, College of Nursing Science, Kyung Hee University, Seoul 02447, Republic of Korea; yj129@khu.ac.kr; 6 Center for Digital Health, Medical Science Research Institute, Kyung Hee University School of Medicine, Kyung Hee University Medical Center, Seoul 02447, Republic of Korea; 7 Department of Precision Medicine, Graduate School, Kyung Hee University, Seoul 02447, Republic of Korea; 8 Department of Convergence Medicine, College of Medicine, Kyung Hee University, Seoul 02447, Republic of Korea

**Keywords:** reactive oxygen species, free radicals, antioxidants, hearing loss

## Abstract

Objective: Although the etiologies and pathogenesis of common hearing disorders—noise-induced hearing loss (NIHL), age-related hearing loss (ARHL), and idiopathic sudden sensorineural hearing loss (ISSNHL)—are diverse, accumulating evidence indicates that reactive oxygen species (ROS) contribute to hearing loss and that antioxidants may help prevent or treat it. We conducted a literature review to examine the relationship between hearing loss and ROS/free radicals in both humans and animal models. Methods: We performed a comprehensive literature search of PubMed/MEDLINE, Embase, the Cochrane Library, Scopus, and Google Scholar to evaluate the induction and role of ROS in the development and treatment of hearing loss. Results: We synthesized evidence across NIHL, ARHL, and ISSNHL. Factors and reactive species implicated in hearing loss included cytomegalovirus infection, genetic polymorphisms, NADPH oxidase 4 (NOX4), NOX transgenic models (NOX-Tg), lipid hydroperoxides (LOOH), and malondialdehyde (MDA). Antioxidant strategies examined for prevention or treatment included vitamins A, C, and E with magnesium; rebamipide; α-lipoic acid; LLY-283; edaravone; melatonin; glutathione peroxidase; superoxide dismutase; glucose; hydrogen-saturated saline; activation of nuclear factor erythroid 2-related factor 2 (Nrf2); inhaled hydrogen gas; and caffeic acid. Conclusions: Elevated ROS and free radicals appear to contribute to the pathogenesis of hearing loss. Although definitive conclusions cannot yet be drawn, current evidence suggests that antioxidant approaches may aid in prevention and treatment. Further studies are needed to elucidate underlying mechanisms, refine therapeutic targets and dosing, and validate efficacy in rigorously designed clinical trials.

## 1. Introduction

### 1.1. Hearing Loss

Hearing loss—one of the most significant symptoms in otologic disease—can arise from dysfunction at any point along the auditory pathway, from the external ear to the auditory cortex. Its etiologies, severity, and sites of involvement are highly heterogeneous. Hearing impairment disrupts language acquisition and development, thereby compromising communication—one of humanity’s greatest assets—and substantially diminishes quality of life [1,2]. Hearing loss can be classified by time of onset into congenital and acquired; by clinical course into progressive and fluctuating; and by site of lesion into conductive, sensorineural, and mixed types. Its severity is typically categorized as normal, mild, moderate, moderately severe, severe, or profound [3].

Except in cases of total deafness (anacusis), hearing loss rarely remains static; it may progress with age or fluctuate depending on the underlying disease. Progressive sensorineural hearing loss in adults can result from age-related hearing loss (presbycusis), noise exposure, hereditary/familial factors, ototoxic medications, or autoimmune mechanisms. Unilateral hearing loss may also be caused by a cerebellopontine angle tumor. Chronic otitis media and otosclerosis typically lead to gradual impairment with conductive or mixed features. Systemic diseases associated with progressive loss include endocrine disorders (e.g., hypothyroidism, diabetes mellitus) and metabolic disorders (e.g., chronic kidney disease, hyperlipidemia). Fluctuating hearing loss can occur with perilymphatic fistula (e.g., after heavy lifting or head trauma) and in autoimmune diseases such as systemic lupus erythematosus and Behçet’s disease. Ménière’s disease presents with recurrent low-frequency hearing loss and episodic vertigo. By site of pathology, hearing impairment is classified into five categories: conductive hearing loss (CHL), sensorineural hearing loss (SNHL), central hearing loss, nonorganic (functional) hearing loss, and mixed hearing loss. CHL arises from lesions of the external or middle ear that impair sound conduction. SNHL results from pathology of the inner ear, which transduces acoustic energy into electrical signals, or of the auditory nerve, which conveys these signals to central pathways. Central hearing loss is due to disorders of the central auditory nervous system, whereas nonorganic hearing loss refers to apparent impairment without identifiable organic pathology. Mixed hearing loss denotes the coexistence of conductive and sensorineural components [4].

According to the Global Burden of Disease study, the prevalence of hearing loss increased from 14.33% in 1990 to 18.06% in 2015, and hearing loss ranked as the fifth leading cause of disability in both developed and developing countries [5]. An analysis of National Health and Nutrition Examination Survey (NHANES) data from 1999–2004 reported that approximately 16.1% of U.S. adults had speech-frequency hearing loss (bilateral 7.3%, unilateral 8.9%; pure-tone average ≥25 dB HL at 0.5, 1, 2, and 4 kHz) and 31% had high-frequency hearing loss (bilateral 19%, unilateral 12%; thresholds ≥25 dB HL at 3, 4, and 6 kHz) [6]. Prevalence rises markedly with age: 3.1% at 20–29 years, 5.4% at 30–39 years, 15.0% at 40–49 years, 29% at 50–59 years, and 49% at 60–69 years [6]. Approximately half of individuals aged 70 years and 80% of those aged 85 years and older have hearing loss severe enough to impair daily communication and social participation. The pathophysiology of hearing loss is complex and multifactorial. Genetic susceptibility, inflammatory processes, systemic diseases, noise exposure, ototoxic medications, oxidative stress, and aging all contribute to risk [7]. With increasing recognition of ROS-driven mechanisms, potential links between ROS and hearing loss have drawn growing attention. Accordingly, this review synthesizes evidence on the roles of ROS and free radicals across major types of hearing loss and their contributions to disease pathogenesis.

### 1.2. Free Radical

Free radicals are unstable atoms or molecules that contain one or more unpaired electrons in their outer orbitals. Because they tend to acquire electrons from other molecules to achieve stability, they are highly reactive and readily engage in chemical reactions with surrounding substrates. Free radicals are generated naturally during physiological processes such as cellular respiration, inflammation, and immune responses, and they can also be induced by environmental stimuli. Importantly, free radicals are not solely harmful; they participate in essential physiological functions. During host defense, for example, macrophages and neutrophils generate species such as superoxide (O_2_∙−), hydrogen peroxide (H_2_O_2_), and hydroxyl radicals (∙OH) via enzymes including NADPH oxidase and myeloperoxidase, thereby oxidizing and damaging microbial membranes, DNA, and proteins. Nitric oxide (NO∙) also acts as a signaling radical, activating transcriptional programs involved in vasodilation, neurotransmission, and immune regulation. In addition, free radicals can promote the removal of damaged cells by triggering apoptotic signaling, contributing to tumor suppression. However, excessive generation of free radicals or insufficient antioxidant defense leads to oxidative stress, which can cause tissue injury and disease. Reactive oxygen species (ROS) oxidize nucleobases in DNA, inducing base modifications and mutations; the accumulation of such lesions promotes genomic instability and contributes to cancer and neurodegenerative disorders. ROS can also oxidize amino acid side chains, alter protein conformation, inhibit enzyme activity, and disrupt signaling pathways, leading to the accumulation of dysfunctional proteins and diverse pathologies. In the inner ear, outer hair cells are particularly susceptible to ROS; exposure to loud noise or ototoxic drugs leads to excessive ROS accumulation, hair-cell death, and consequent hearing loss. The body employs antioxidant systems to neutralize free radicals and maintain redox homeostasis. Enzymatic antioxidants such as superoxide dismutase, catalase, and glutathione peroxidase mitigate radical-mediated damage by converting reactive species to less harmful products, while repair and clearance pathways resolve oxidized biomolecules. Maintaining the balance between free radical production and antioxidant defenses is therefore essential for sustaining cellular homeostasis and preventing disease [8,9,10,11].

## 2. Research Method

**Figure 1:**
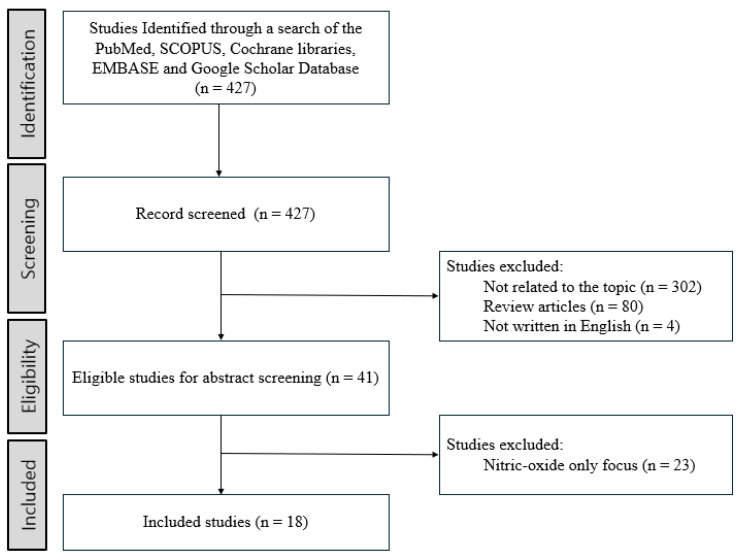
Review flow diagram.

This narrative review followed a prespecified, structured process to synthesize evidence on the roles of free radicals/reactive oxygen species (ROS) in hearing loss across etiologies and interventions. We searched PubMed/MEDLINE, Embase, the Cochrane Library, Scopus, and Google Scholar from 1 January 1988 to 31 January 2025, restricted to English to ensure consistent terminology and indexing. Core concepts and synonyms included “free radical,” “reactive oxygen species,” “oxidative stress,” “hearing loss,” “sensorineural,” “noise-induced,” “age-related/presbycusis,” and “sudden sensorineural hearing loss.” An example PubMed query was: (“free radical” OR “reactive oxygen species” OR “oxidative stress”) AND (“hearing loss” OR sensorineural OR “noise-induced” OR presbycusis OR “sudden sensorineural”). For Google Scholar, we screened the first 200 results sorted by relevance. Inclusion criteria: original human or animal studies evaluating free radicals/ROS in hearing-loss pathogenesis; reporting oxidative biomarkers (e.g., MDA, 4-HNE) and/or antioxidant interventions; and including audiologic and/or histologic outcomes. Exclusion criteria: off-topic articles; review articles, editorials, or conference abstracts without full text; non-English publications; studies focused exclusively on nitric oxide as covered in our prior review; and duplicate or superseded reports. Two reviewers (J.M.L. and Y.J.C.) independently screened titles/abstracts and assessed full texts, resolving disagreements by consensus. We extracted study design, population/species, exposure/intervention, oxidative markers, outcomes, and key findings. Given heterogeneity in models, interventions, and outcome measures, we did not apply a numerical quality score; instead, we conducted a targeted qualitative appraisal across predefined domains: (1) randomization or baseline comparability; (2) blinding of investigators/outcome assessors; (3) validity and consistency of outcome measures; (4) completeness of outcome data and handling of attrition; and (5) selective reporting. These appraisals informed a narrative, theme-based synthesis; a meta-analysis was not undertaken due to substantial clinical and methodological heterogeneity. Of 427 records identified, 386 were excluded at title/abstract screening (302 off-topic; 80 review articles; 4 non-English). Forty-one full texts were assessed; 23 nitric-oxide–only studies were excluded, leaving 18 studies for qualitative synthesis. The updated flow diagram (Figure 1) displays counts and reasons at each step.

## 3. Results

To assess the contribution of ROS to the pathogenesis of hearing loss, we reviewed 18 eligible studies. We categorized studies by whether they supported a deleterious role for ROS in hearing loss or reported no association. Seventeen studies indicated that ROS contribute to the pathogenesis of hearing loss, whereas one study found no relationship between ROS and the underlying pathophysiology.

### 3.1. Studies Identifying Free Radicals and Reactive Oxygen Species as Contributors to Hearing Loss (Table 1)

ROS—including superoxide anion, hydroxyl radical, H_2_O_2_, and singlet oxygen—play key roles in numerous physiological and pathological processes [12]. While appropriate ROS levels are essential for cell survival and differentiation, excessive ROS cause oxidative stress in multiple organs (inner ear, heart, brain, liver, kidney), contributing to SNHL, myocardial infarction, heart failure, neurodegenerative diseases, and hepatic or renal fibrosis [13,14]. Most SNHL results from reduced hearing sensitivity due to cochlear injury and/or hair cell death [15]. The cochlea contains two hair cell types: inner hair cells (IHCs), which detect sound and transmit acoustic information to the brain, and outer hair cells (OHCs), which provide active mechanical amplification for high sensitivity and sharp frequency resolution. Along the tonotopic gradient, the basal turn encodes high frequencies and the apical turn low frequencies. Among inner ear NADPH oxidases, NO is upregulated in the cochlea after cisplatin administration in rodents, driving ROS overproduction and apoptosis in cochlear cells, including OHCs. Small interfering RNA (siRNA) targeting NO mitigates OHC damage and hearing deterioration. In mice, cisplatin exposure also increases expression of NADPH oxidase 4 (NOX4) and NOX1, indicating that ROS mediated vulnerability to SNHL may depend on the specific NOX isoforms engaged by a given insult. To investigate ROS induced SNHL—particularly noise-induced hearing loss (NIHL)—researchers generated a transgenic mouse line overexpressing human NOX4-Tg as an in vivo model of ROS excess. Although ROS overproduction was evident in the cochleae of NOX4-Tg mice, baseline hearing was normal. After intense noise exposure, however, these mice exhibited increased hearing vulnerability, most prominently at high frequencies, accompanied by OHC loss in the basal turn. The pattern suggested that OHCs are primary targets of acute ROS surges. Notably, both the hearing deficit and OHC loss were rescued by the antioxidant Tempol. In complementary cell models (HEK293 cells with stable or transient NOX4 expression, or treated with H_2_O_2_), heat shock protein 47 (HSP47) levels increased. Consistently, Hsp47 was upregulated in both the cochlea and heart of NOX4-Tg mice. These findings suggest that Hsp47 may function as an endogenous protective factor induced by chronic ROS exposure, helping to buffer ROS related cochlear injury. In summary, NOX4-Tg mice maintain normal baseline hearing but show heightened susceptibility after strong noise exposure, consistent with adaptive activation of antioxidant pathways under chronic ROS excess [5,16].

**Table 1:** Chemistry-based classification of bioactive compounds and their mechanisms/benefits in hearing-loss prevention.

Chemical Property/Class	Representative Compounds	Mechanism or Benefit
Hydrophilic antioxidants	Vitamin C	Scavenges radicals in aqueous phase; complements membrane antioxidants
Lipophilic antioxidants	Vitamin E; Vitamin A/β-carotene	Chain-breaking antioxidant; interrupts lipid peroxidation in membranes; quenches singlet oxygen
Amphipathic antioxidants	α-Lipoic acid	Redox-active cofactor; supports antioxidant network (reported benefit in ARHL regimen with vitamin C)
Polyphenols	Caffeic acid	Attenuates superoxide and 4-HNE; preserves ABR/SGN after noise exposure
Gaseous/small-molecule reductants	H_2_	Reduces oxidative injury; improves thresholds and OHC survival after noise
Free-radical scavengers (small molecules)	Edaravone	Hydroxyl-radical scavenger; protects cochlea in ischemia models; mixed ISSNHL clinical benefit
Antioxidant enzyme systems/inducers	SOD, GPx; Melatonin	Endogenous ROS detox; melatonin prevents MDA rise and preserves thresholds under noise
Redox-response pathway activators	Nrf2 activators	Upregulate cytoprotective/antioxidant genes; reduce NIHL vulnerability

Abbreviations: 4-HNE, 4-hydroxy-2-nonenal; ABR, auditory brainstem response; ARHL, age-related hearing loss; GPx, glutathione peroxidase; H_2_, hydrogen; ISSNHL, idiopathic sudden sensorineural hearing loss; MDA, malondialdehyde; NIHL, noise-induced hearing loss; Nrf2, nuclear factor erythroid 2-related factor 2; OHC, outer hair cell; ROS, reactive oxygen species; SGN, spiral ganglion neuron; SOD, superoxide dismutase.

#### 3.1.1. NOX4-Tg Versus Piccolo 1 and CtBP2

ROS play central roles in diverse physiological and pathological processes. While appropriate ROS levels are essential for cell survival and differentiation, elevated ROS induce oxidative stress in multiple organs, including the inner ear and central nervous system (CNS), leading to disorders such as SNHL, CNS injury, and neurodegeneration [14]. Prior studies implicate ROS in acquired forms of SNHL—age-related hearing loss (ARHL), NIHL, and drug-induced hearing loss (DIHL). Although ROS contribute to cochlear hair-cell (HC) loss in these conditions, the molecular links between ROS and HC degeneration, as well as between ROS and cochlear synaptopathy, remain incompletely defined. To interrogate these relationships, investigators used NOX4 transgenic (NOX4-Tg) mice that constitutively generate ROS. Male and female NOX4-Tg mice carrying a genomic integration of a 17-gene CAG promoter–3xFLAG-NOX4–polyA cassette were compared with age- and sex-matched wild-type (WT) littermates. To assess the impact of ROS on synaptic ribbon components, WT mice were exposed at postnatal week 2 to intense noise, a known inducer of ROS. In NOX4-Tg cochleae at postnatal day 6, mRNA levels of Piccolo 1—a key synaptic ribbon component—were reduced relative to WT, consistent with synaptic ribbons being ROS targets. Another ribbon component, C-terminal binding protein 2 (CtBP2), was significantly decreased in NOX4-Tg cochleae at 1 and 4 months of age, whereas no significant difference was observed at 1.5 and 2 months. CtBP2 reduction plateaued by 4 months in NOX4-Tg mice but declined progressively from 1 to 6 months. Moreover, in 2-month-old NOX4-Tg mice, CtBP2 levels decreased significantly after cisplatin treatment or noise exposure compared with WT. Collectively, the observed reductions in synaptic ribbon components (Piccolo 1 mRNA at P6 and CtBP2 protein at 1 month and ≥4 months) in NOX4-Tg mice indicate that ROS may contribute both to delayed development and to early degeneration of ribbon synapses in the cochlea [17].

#### 3.1.2. NOS3 Polymorphism

Despite extensive research, the etiologies of idiopathic sudden sensorineural hearing loss (SSNHL) and Ménière’s disease (MD) remain incompletely understood. Emerging evidence implicates free radicals in inner-ear pathology, and genetic factors may also contribute to susceptibility to SSNHL and MD [18,19]. Reported associations in SSNHL involve polymorphisms in genes related to vascular, circulatory, or inflammatory pathways, including protein kinase C eta (1425G/A), matrix metalloproteinase-1 (−1607G/2G), interleukin-1A (−889C/T), interleukin-6 (−572C/G), methylenetetrahydrofolate reductase (MTHFR C677T), prothrombin (G20210A), platelet glycoprotein IIIa (A1/A2), factor V Leiden, and complement factor H [20]. In MD, significant associations have been reported for polymorphisms in genes such as KCNE (in Japanese but not Caucasian populations), adducin 1 (Gly460Trp), heat-shock protein 70-1 (190G/C), interleukin-1A (−889C/T), AQP5, and MTHFR (C677T). A case–control study in Japan examined seven polymorphisms in genes implicated in free-radical/redox pathways—methionine synthase (MTR; rs1805087), methionine synthase reductase (MTRR; rs1801394), nitric oxide synthase 3 (NOS3; rs1799983), caveolin-1 (CAV1; rs3840634), melatonin receptor 1B (MTNR1B; rs1387153), the NADPH oxidase p22^phox^ subunit (CYBA; rs4673), and mitochondrial 5178 (MT5178; rs28357984)—in 83 patients with SSNHL and 83 with MD. Controls were drawn from the National Institute for Longevity Sciences–Longitudinal Study of Aging (NILS-LSA) cohort (*n* = 2048 for SSNHL comparisons; *n* = 1946 for MD comparisons). NOS3 rs1799983 was significantly associated with increased risk of SSNHL, and CAV1 rs3840634 was significantly associated with MD risk. Thus, in this Japanese cohort, NOS3 (rs1799983) polymorphism correlated with SSNHL susceptibility, whereas CAV1 (rs3840634) polymorphism correlated with susceptibility to Ménière’s disease [21].

#### 3.1.3. ROS Versus Antioxidants

Ischemia–reperfusion injury of the cochlea is a proposed mechanism of idiopathic sudden sensorineural hearing loss (ISSNHL), and oxidative stress has recently been implicated as a risk factor for microvascular damage. To evaluate the putative role of oxidative stress in ISSNHL, serum ROS levels and total antioxidant capacity (TAC) were measured by spectrophotometry using a F.R.E.E. analyzer (Diacron International, Grosseto, Italy) in 39 patients with ISSNHL and 70 healthy controls. An overall oxidative stress index integrating both oxidant and antioxidant status was also calculated. Of 39 patients, 25 exhibited oxidative stress, reflected by significantly higher ROS levels than controls (348.2 ± 84.8 vs. 306.75 ± 46.7 UCarr; *p* = 0.001). The oxidative stress index was likewise higher in patients than controls (0.75 ± 2.4 vs. −0.0007 ± 1.28 AU; *p* = 0.03). In contrast, TAC did not differ significantly between groups (442.6 ± 80.7 vs. 433.5 ± 71.9 μmol HClO/mL). Overall, the oxidant index (Oxy-I) was increased in patients relative to controls (0.75 ± 2.4 vs. −0.0007 ± 1.28 AU; *p* = 0.03). These findings support oxidative stress as a major determinant of endothelial dysfunction, suggesting that microvascular injury contributes to the pathogenesis of ISSNHL [22].

#### 3.1.4. 4-HNE Versus LLY-283

In a mouse model of NIHL, investigators evaluated the effect of LLY-283, a selective inhibitor of protein arginine methyltransferase 5 (PRMT5). Eight-week-old male C57BL/6 mice were exposed in a small reverberant chamber to broadband noise (1–20 kHz) at 120 dB SPL for 2 h. Two days after exposure, auditory brainstem responses (ABRs) were recorded to assess permanent threshold shifts (PTS), and cochlear hair-cell loss was quantified. As reported previously, noise increased ROS levels, which can activate multiple apoptotic pathways [23]. Because oxidative stress is a key driver of NIHL, cochlear expression of 4-hydroxy-2-nonenal (4-HNE), a lipid peroxidation marker, was examined. 4-HNE was elevated in noise-exposed cochleae compared with controls. Notably, pretreatment with LLY-283 markedly attenuated the noise-induced increase of 4-HNE in hair cells and significantly preserved spiral ganglion neurons (SGNs). These findings suggest that PRMT5 inhibition with LLY-283 mitigates cochlear oxidative damage and hair-cell apoptosis following acoustic overexposure [24].

#### 3.1.5. Malondialdehyde Versus Glutathione Peroxidase

To evaluate noise-related hearing damage in industrial workers, investigators measured malondialdehyde (MDA) as a marker of free-radical lipid peroxidation and glutathione peroxidase (GSH-P) activity as an antioxidant marker. Sixty male employees at a hydroelectric power plant were assigned to three exposure groups with differing continuous noise levels: Group I (turbine; 95–110 dB SPL, 8 h/day without hearing protection; *n* = 20), Group II (machinery maintenance workshop; 75–85 dB SPL, 8 h/day without protection; *n* = 20), and Group III (outdoor; ≤75 dB SPL; *n* = 20). A control group comprised 20 male volunteers employed at the same medical center. Pure-tone thresholds were measured at seven frequencies from 250 to 8000 Hz. Blood samples (10 mL) were collected to determine plasma MDA and erythrocyte GSH-Px activity. The 60 exposed workers (mean exposure duration 14.21 ± 6.11 years; age 28–49 years, 37.7 ± 5.6) were comparable to controls (age 28–48 years, 36.8 ± 4.5). Mean pure-tone thresholds were significantly higher in Groups I and II than in Group III and controls across 2–8 kHz. Mild sensorineural hearing loss was observed at 4 kHz in Group I and at 6 kHz in Group II. Compared with controls, both Groups I and II showed statistically significant differences (*p* < 0.05). MDA levels were elevated in exposed workers versus controls, reaching significance in Group I only (*p* < 0.05). Erythrocyte GSH-Px activity was significantly increased in Groups I and II compared with the other groups (*p* < 0.05). These findings suggest that reactive oxygen species contribute to noise-related hearing impairment, as reflected by increased lipid peroxidation and compensatory upregulation of antioxidant enzyme activity [25].

#### 3.1.6. LOOH and Thiols

A prospective study evaluated oxidative stress status in patients with prelingual profound SNHL and assessed the impact of cochlear implantation on redox markers. Group 1 comprised 25 patients with bilateral congenital profound SNHL scheduled for cochlear implantation; Group 2 included the same patients 6 months post-implantation (serum collected at follow-up); Group 3 consisted of 25 healthy controls. Serum samples were obtained 2 weeks before surgery and 6 months after surgery in the patient cohort, and once in controls. Total thiol, native thiol, disulfide, and lipid hydroperoxide (LOOH) levels were measured. Before surgery, patients with SNHL had significantly lower native thiol (*p* < 0.01) and total thiol (*p* < 0.01) levels and significantly higher LOOH levels (*p* < 0.04) than controls. After implantation, native and total thiols increased and LOOH decreased relative to preoperative values, but these changes did not reach statistical significance. These findings indicate that individuals with congenital profound SNHL are in a state of oxidative stress; however, cochlear implantation did not produce a significant amelioration of systemic oxidative stress at 6 months postoperatively [26].

#### 3.1.7. Cytomegalovirus and Vitamins

Cytomegalovirus (CMV) infection is the most common congenital infection in the United States and a leading nongenetic cause of SNHL. Although excessive ROS have been implicated in several forms of hearing loss—including noise-induced, ototoxic, and age-related loss [27]—the injury mechanisms underlying CMV-related hearing loss and the role of ROS have been less well characterized. In a neonatal mouse model, inbred BALB/c and hybrid-background Nrf2^−/−^ pups were inoculated intracerebrally with CMV on postnatal day 3; controls received an equal volume of saline. ROS generation was assessed by dihydroethidium (DHE) fluorescence. Two antioxidant regimens—D-methionine (D-Met; Sigma-Aldrich) and a combination of vitamins A, C, and E with magnesium (ACE-Mg)—were administered beginning 1 h before infection and continued for 14 days. Infected Nrf2^−/−^ mice exhibited significantly worse hearing than uninfected controls (*p* < 0.001). By 7 days post-inoculation, DHE fluorescence was significantly increased in CMV-infected BALB/c mice compared with uninfected littermates. Antioxidant treatment with D-Met or ACE-Mg reduced DHE fluorescence and significantly improved auditory brainstem response (ABR) and distortion-product otoacoustic emission (DPOAE) thresholds relative to untreated infected mice (*p* < 0.0001). Scanning electron microscopy demonstrated less outer hair-cell loss in antioxidant-treated infected mice than in untreated infected mice. These findings indicate that excessive ROS contribute to CMV-induced hearing loss and that antioxidant therapy can mitigate cochlear injury and functional deficits following CMV infection [28].

#### 3.1.8. Vitamins Plus Magnesium

Free-radical generation within the cochlea plays a pivotal role in NIHL. The amount, spatial distribution, and time course of radical production are relevant; clinically meaningful levels of reactive oxygen and nitrogen species are generated after acoustic overexposure, and free-radical formation has been linked to reduced cochlear blood flow. To test whether antioxidants prevent NIHL, male guinea pigs (250–300 g) with normal Preyer’s reflexes were assigned to four groups. All groups received once-daily treatment beginning 1 h before noise exposure and continuing at 24-h intervals for a total of six doses through day 5 after exposure to octave-band noise (center frequency 4 kHz, 120 dB SPL, 5 h). Control (*n* = 9): intraperitoneal saline (1 mL). ACE (*n* = 8: vitamin A (β-carotene 2.1 mg/kg, oral), vitamin C (L-threoascorbic acid 71.4 mg/kg, subcutaneous), and vitamin E (Trolox 26 mg/kg, subcutaneous). Mg (*n* = 6): magnesium sulfate 2.85 mmol/kg (343 mg/kg), subcutaneous. ACE + Mg: combined administration of the above antioxidants and magnesium. ABR thresholds measured after exposure were significantly lower in the ACE + Mg group than in all other groups (all *p* < 0.001), indicating superior preservation of hearing. These findings support roles for both free-radical generation and noise-induced vasoconstriction in the onset and progression of NIHL, and demonstrate that vitamins A, C, and E act synergistically with magnesium to prevent acoustic trauma. Each agent has a distinct mode of action. β-Carotene (converted in vivo to vitamin A) quenches singlet oxygen, thereby preventing formation of lipid hydroperoxides from membrane lipids. Vitamin E, a lipophilic chain-breaking antioxidant residing in cellular membranes, reduces peroxyl radicals and interrupts the propagation phase of lipid peroxidation. Vitamin C scavenges radicals in the aqueous phase, complementing vitamin E’s membrane-localized effects [29,30]. Owing to differences in chemical environment and targets, hydrophilic and lipophilic antioxidants exhibit complementary and synergistic radical-scavenging actions [30].

#### 3.1.9. Rebamipide, α-Lipoic Acid, and Vitamin C

To evaluate whether radical-scavenger therapy could be an effective treatment for age-related hearing loss, 46 patients (10 men, 36 women; mean age 76.7 years, range 70–91) with age-related hearing impairment and no other identifiable causes received rebamipide (300 mg/day), vitamin C (600 mg/day), and α-lipoic acid (60 mg/day) for at least 8 weeks. Pure-tone thresholds at 125, 250, 500, 1000, 2000, 4000, and 8000 Hz were measured before treatment and at 8 weeks. Pretreatment hearing levels were compared with the final hearing level after eight weeks of treatment. Analysis by frequency of the study results showed that at 125 Hz, 40 ears (43.5%) showed clinically significant hearing improvement (≥10 dB), 49 ears showed no change, and 3 ears showed worsening. At 250 Hz, 38 ears (41.3%) showed improvement, 50 ears showed no change, and 4 ears showed worsening. At 500 Hz, 34 ears (37.0%) showed improvement, 56 ears showed no change, and 2 ears showed worsening. At 1000 Hz, 22 ears (23.9%) showed improvement, 64 ears showed no change, and 6 ears showed worsening. At 2000 Hz, 9 ears (9.8%) showed improvement, 78 ears showed no change, and 5 ears showed worsening. At 4000 Hz, 21 ears (22.8%) showed improvement, 68 ears showed no change, and 3 ears showed worsening. At 8000 Hz, 34 ears (37.0%) improved, 55 ears showed no change, and 3 ears showed deterioration. The mean pure-tone average improved from 42.5 ± 7.87 dB pretreatment to 38.2 ± 8.06 dB after therapy (*p* < 0.001). Significant threshold changes were observed across 125–8000 Hz. These findings suggest that a regimen of rebamipide, vitamin C, and α-lipoic acid has therapeutic potential for age-related hearing loss; however, the short follow-up warrants longer and broader evaluations to confirm durability and preventive effects [31].

#### 3.1.10. Edaravone

Edaravone (MCI-186; 3-methyl-1-phenyl-2-pyrazolin-5-one) is a novel free-radical scavenger approved clinically for ischemic brain injury. It suppresses hydroxyl radicals and attenuates iron-induced peroxidative injury [32,33]. To evaluate its therapeutic potential for human sensorineural hearing loss, adult male Mongolian gerbils (Meriones unguiculatus; 60–80 g) were assigned to three groups: (1) ischemic animals treated with vehicle (saline), (2) ischemic animals treated with edaravone, and (3) non-ischemic (sham-operated) animals treated with edaravone. Cochlear ischemia was induced by bilateral vertebral artery occlusion for 15 min, followed 1 h later by intravenous administration of edaravone (1 mg/kg) or saline. In vehicle-treated ischemic animals, the auditory brainstem response (ABR) threshold shift was 24.1 dB and IHC counts decreased by 26.5%. In contrast, edaravone-treated ischemic animals showed a markedly smaller ABR threshold shift (7.5 dB and only an 8.8% loss of IHCs. These findings indicate that edaravone protects the cochlea from transient ischemic injury and support a key role for free radicals in ischemia-related cochlear damage, suggesting that free-radical scavengers may be useful in the treatment of sensorineural hearing loss [33].

#### 3.1.11. Melatonin and Methylprednisolone

Melatonin, secreted by the pineal gland in a circadian manner, exerts direct antioxidant effects, powerfully scavenging free oxygen radicals (FORs) and enhancing the activity of the antioxidant enzyme glutathione peroxidase (GSH-Px) [34]. High-dose methylprednisolone has shown antioxidant properties in vitro, preventing FOR-mediated lipid membrane damage and significantly reducing MDA levels after ischemia–reperfusion, thereby limiting tissue injury [35]. To examine cochlear injury induced by noise-generated FORs and to test the prophylactic effects of melatonin and methylprednisolone, fifty male albino guinea pigs were randomly assigned to five groups. Group I received neither noise exposure nor treatment. All other groups were exposed to continuous broadband noise at 100 ± 2 dB for 60 h. Group II received noise only (no drug). Group III received noise plus melatonin. Group IV received noise plus methylprednisolone. Group V received noise plus both agents. Methylprednisolone (40 mg/kg) and/or melatonin (20 mg/kg) were administered intramuscularly 24 h before noise, immediately before noise, and once daily until the end of exposure. Immediately after noise exposure, animals were decapitated and venous blood was collected into EDTA tubes; plasma MDA, erythrocyte GSH-Px activity, and cochlear tissue MDA were measured. Compared with Group I, Group II showed increased plasma and cochlear tissue MDA, decreased erythrocyte GSH-Px activity, and elevated auditory thresholds (all *p* < 0.01). Significant differences in MDA and erythrocyte GSH-Px were observed between Groups II and III (*p* < 0.01), indicating protection with melatonin. In contrast, auditory thresholds and MDA levels in Groups IV and V were similar to Group II (*p* > 0.05). In conclusion, methylprednisolone alone—or in combination with melatonin under this dosing paradigm—did not provide adequate prophylaxis against noise-induced cochlear damage, whereas melatonin alone prevented the rise in MDA and the fall in erythrocyte GSH-Px, thereby preserving hearing thresholds. Melatonin may therefore represent a promising, effective, and reliable alternative for the prevention of NIHL [36].

#### 3.1.12. Glucose

Oxidative stress is a major determinant in the pathogenesis of NIHL. Because cellular defenses against oxidative stress are energy-consuming, this study tested whether glucose supplementation, by increasing energy availability, could protect cochlear hair cells and mitigate NIHL. Eight-week-old CBA/J mice underwent baseline ABR testing at 9 weeks and were exposed at 10 weeks to broadband noise (2–20 kHz) at 115 dB SPL for 2 h. To assess protection, mice received an intraperitoneal injection of glucose (4.5 or 9.0 g/kg) 30 min before noise; controls received saline. Cochlear oxidative stress was monitored by immunolabeling for 4-HNE and 3-nitrotyrosine (3-NT). One hour after noise exposure, OHC immunolabeling for 4-HNE (t(6) = −4.3, *p* = 0.013) and 3-NT (t(6) = −6.102, *p* = 0.004) was increased. Glucose supplementation significantly attenuated the noise-induced elevations of 4-HNE (t(6) = 3.104, *p* = 0.036) and 3-NT (t(6) = 4.628, *p* = 0.01), and reduced loss of outer hair cells, inner hair-cell synaptic ribbons, and ABR threshold shifts. These findings indicate that glucose can lessen noise-induced oxidative stress in the cochlea and mitigate NIHL [37].

#### 3.1.13. Hydrogen

To determine whether hydrogen-saturated saline protects against hearing loss induced by intense narrow-band noise, guinea pigs were assigned to three groups: (1) hydrogen-saturated saline (1 mL/100 g, intraperitoneal) administered once daily for 3 days before noise and again 1 h before exposure (*n* = 20); (2) normal saline (0.9%) given on the same schedule as a control (*n* = 20); and (3) untreated, unexposed animals as normal controls (*n* = 15). Hearing was evaluated by auditory brainstem responses (ABR) and distortion-product otoacoustic emissions (DPOAEs). Cochlear free-radical changes were assessed before noise, immediately after exposure, and 7 days after exposure. Immediately after narrow-band noise exposure (130 dB SPL, 1 h), the mean ABR threshold shift was 54 dB SPL in the hydrogen-saline group versus 62 dB SPL in the normal-saline group. DPOAE amplitudes decreased markedly from 1–8 kHz in both noise-exposed groups. Seven days after noise, DPOAE amplitudes in the hydrogen-saline group were less reduced than in the saline group across 0.5–4 kHz. By 14 days, hydrogen-saline–treated animals showed significantly higher DPOAE amplitudes than saline controls at 0.5, 0.75, 1, 2, 3, and 4 kHz. Pretreatment with hydrogen-saturated saline substantially reduced noise-induced hair-cell damage and hearing loss. Biochemically, malondialdehyde, lipid peroxidation, and hydroxyl radical levels were significantly lower after acoustic trauma in the hydrogen-saline group, indicating attenuation of harmful free radicals. These results suggest that hydrogen-saturated saline effectively prevents intense narrow-band noise–induced hearing loss via antioxidant mechanisms [38].

#### 3.1.14. Nrf2 (Nuclear Factor Erythroid 2-Related Factor 2)

NIHL is among the most common forms of sensorineural impairment. Recent studies indicate that its pathogenesis is closely linked to cochlear ischemia–reperfusion injury, driven by noise-evoked reductions in blood flow and excessive free-radical generation [39]. This suggests that enhancing cochlear defenses against oxidative stress is a rational therapeutic strategy. Nrf2 is a transcriptional activator that orchestrates antioxidant and cytoprotective responses. Using wild-type and Nrf2-deficient (Nrf2^−/−^) mice on a C57BL/6 background, investigators exposed animals to octave-band noise (8–16 kHz) at 96 dB SPL for 2 h to assess Nrf2’s contribution to cochlear protection. Seven days after exposure, ABR threshold shifts were significantly larger in Nrf2^−/−^ mice than in wild-type controls, indicating heightened susceptibility. Pretreatment with the potent Nrf2 activator 2-cyano-3, 12-dioxooleana-1, 9-dien-28-imidazolide (CDDO-Im) preserved hair-cell integrity, reduced oxidative stress after exposure, and improved hearing thresholds in wild-type mice, but not in Nrf2^−/−^ mice, which also exhibited a significant decrease in glutathione (GSH). These findings demonstrate that Nrf2 activation confers protection against NIHL and support Nrf2 as a preventive therapeutic target [40].

#### 3.1.15. Caffeic Acid

Imbalance of cellular redox status and activation of inflammatory pathways are considered key, shared mechanisms in NIHL. Focusing on caffeic acid (CA)—a polyphenolic hydroxycinnamic acid [41,42]—researchers evaluated its protective effects in a NIHL model. Adult male Wistar rats (200–250 g; ~2 months old; *n* = 113) were assigned to six primary groups: (1) normal-hearing controls (*n* = 25); (2) noise-exposed animals (pure tone, 10 kHz, 120 dB SPL, 60 min; *n* = 25); (3) noise-exposed animals treated with CA (30 mg/kg, i.p.) 1 h before noise and once daily for 3 consecutive days thereafter (Noise + CA; *n* = 25); (4) control + CA (*n* = 10); (5) control + DMSO vehicle (*n* = 14); and (6) noise + DMSO (*n* = 14). To define an optimal dose, additional noise-exposed rats (*n* = 10 each) received CA at 15 or 50 mg/kg using the same schedule. Auditory brainstem response (ABR) thresholds were measured longitudinally. Baseline ABRs (day 0) did not differ among groups. In the noise group, mean threshold shifts increased markedly by day 1, reaching ~45–50 dB at 12–24 kHz, followed by a gradual partial recovery (~5–10 dB by day 3. A permanent threshold shift of ~30–35 dB was evident at day 7 and persisted through day 21. CA at 30 mg/kg significantly attenuated threshold shifts across all frequencies at every time point: day-1 shifts were ~25–30 dB, with a further ~5–10 dB improvement by day 3; no additional change occurred between days 3 and 7. Protection was most pronounced in the mid-to-high frequencies—those most vulnerable to the noise exposure—reducing noise-induced shifts by ~20–25 dB. In contrast, 15 mg/kg and 50 mg/kg CA did not confer measurable protection.

Mechanistic assays showed increased DHE fluorescence in SGNs, the organ of Corti (OC), and the stria vascularis (StV) within the middle–basal turn on day 1 after noise, with further superoxide formation detected through days 3–7. CA (30 mg/kg) significantly reduced superoxide signals across all cochlear structures from day 1 to day 7, counteracting redox imbalance. Regarding lipid peroxidation, low baseline 4-HNE labeling in controls increased in hair cells, SGNs, and StV by day 1 after noise, expanded within StV by day 3, and peaked across all structures by day 7. CA treatment markedly decreased 4-HNE fluorescence at all time points. These data indicate that caffeic acid at 30 mg/kg mitigates NIHL by dampening superoxide production and lipid peroxidation and by targeting both inflammatory signaling and cochlear redox imbalance, supporting CA as a promising therapeutic candidate for noise-related cochlear injury [43].

#### 3.1.16. Inhaled Hydrogen (H_2_)

Molecular H_2_ has emerged as a potential antioxidant capable of reducing ROS [44]. To test whether inhaled H_2_ can prevent NIHL, female Hartley guinea pigs were exposed to one-octave band noise centered at 4 kHz (121 dB SPL, 5 h/day) for 5 consecutive days. During each exposure day, animals inhaled H_2_ for 5 h at concentrations of 0.5%, 1.0%, or 1.5%. Thirty guinea pigs were assigned to 0.5% H_2_ (*n* = 6), 1.0% H_2_ (*n* = 6), 1.5% H_2_ (*n* = 6), or untreated noise-exposed controls (*n* = 6) and were sacrificed 1 week after the final exposure; an additional normal (unexposed, untreated) group (*n* = 6) was also studied. Threshold shifts were improved in the 1.0% and 1.5% H_2_ groups compared with untreated controls. In the basal cochlear turn, OHC survival was significantly higher in the 1.0% and 1.5% H_2_ groups than in controls. Immunohistochemistry for 8-hydroxy-2′-deoxyguanosine (8-OHdG), a marker of oxidative DNA damage, showed strong labeling in untreated noise-exposed animals but reduced labeling in H_2_-treated groups. These findings indicate that free radicals play a critical role in NIHL and that inhaled hydrogen gas mitigates oxidative injury. Inhaled H_2_ may therefore represent a promising therapeutic approach for acute sensorineural hearing loss, including age-related, noise-induced, and drug-induced forms [45] (Table 2).

### 3.2. Studies Reporting an Uncertain or No Etiologic Role of Free Radicals/ROS in Hearing Loss (Table 3)

#### Edarovone

While most studies report that free radicals and reactive oxygen species contribute to hearing loss, one study found an uncertain or no association. Edaravone, a free-radical scavenger widely used in Japan since 2001 to reduce neuronal injury after acute cerebral infarction [46,47], was evaluated in patients with ISSNHL with severe baseline impairment. Fourteen ISSNHL patients with mean hearing levels ≥ 90 dB received edaravone and were compared with 14 propensity-matched controls selected from 45 patients who had similar prognostic factors and underwent hyperbaric oxygen (HBO) therapy. Final hearing levels in the edaravone group were slightly better distributed than in controls, but HBO co-administration within the edaravone group did not clearly influence outcomes, and there was no difference between 30 mg and 60 mg/day dosing. Although the distribution of recovery grades favored the edaravone group, the mean degree of hearing recovery did not differ significantly between groups (paired *t*-test, *p* = 0.80). Overall, final hearing outcomes were essentially comparable between edaravone and control groups, and adding edaravone conferred no clear advantage over prior regimens including HBO. The authors concluded that, despite suggesting that edaravone might substitute for HBO in some settings, edaravone did not produce a significant improvement in hearing recovery compared with existing ISSNHL therapies [48].

**Table 3:** Studies reporting an uncertain or no etiologic role of free radicals/ROS in hearing loss.

Author [Reference]	Study Design	Species and/or Sample	Detection Method	Target Gene(s)	Results/Conclusions
Sano H, et al., 2010 [48]	Clinical study	28 patients	Pure-tone audiometry	N/A	No statistically significant difference in final hearing levels between edaravone and control (hyperbaric oxygenation therapy; HBO) group. Edaravone showed wide variation in outcomes, with some patients recovering well and others showing little improvement. Edaravone did not show a significant advantage over hyperbaric oxygen therapy in the treatment of ISSHL with profound hearing loss. However, due to its safety and ease of use, edaravone may be considered as a potential alternative to HBO, especially for patients with poor prognosis.

### 3.3. Mechanistic Links Among ROS, Cochlear Injury, and Antioxidant Protection

Across models, noise and other insults elevate lipid-peroxidation markers (e.g., 4-HNE, MDA) and superoxide in hair cells, spiral ganglion neurons, and the stria vascularis, paralleling threshold shifts and cellular loss. In NOX4-Tg mice, chronic ROS priming increases noise vulnerability at high frequencies, which is rescued by Tempol; HSP47 upregulation indicates an endogenous stress response. Pharmacologic Nrf2 activation with CDDO-Im reduces oxidative damage and preserves thresholds after noise, consistent with a cytoprotective transcriptional program. Combined ascorbate plus magnesium (ACE + Mg) provides synergistic protection via phase-specific scavenging and vasoregulatory effects. Melatonin prevents MDA increases and preserves thresholds under conditions where methylprednisolone does not. Caffeic acid attenuates superoxide and 4-HNE while preserving ABR thresholds and SGNs, and inhaled H_2_ limits oxidative injury, improving OHC survival and thresholds. Similar oxidative signatures and antioxidant responsiveness are observed in CMV-related hearing loss, where D-methionine or ACE-Mg reduce ROS and improve auditory outcomes. Together, these data link ROS surges to cochlear injury and demonstrate mechanism-consistent protection by antioxidant or redox-modulating strategies.

### 3.4. Conflicting Findings and Variability in Therapeutic Response

Although edaravone reduces cochlear injury in ischemia models, clinical data in ISSNHL show no significant improvement in mean hearing recovery versus controls, despite a favorable distribution of recovery grades; neither co-administration with hyperbaric oxygen nor dosing differences (30 vs. 60 mg/day) altered this result. These discrepancies likely reflect etiologic heterogeneity (ischemic vs. idiopathic subtypes), timing of intervention relative to the oxidative peak, and variability in background therapies, underscoring the need for stratified trial designs and standardized protocols. Target/pathway specificity also matters: direct radical scavengers, transcriptional activators (e.g., Nrf2), and anti-inflammatory agents are not necessarily interchangeable across etiologies or therapeutic windows. For example, PRMT5 inhibition reduces 4-HNE and preserves SGNs after noise, but its comparative effectiveness versus other antioxidant strategies remains undefined. Considering temporality, consistency, biological plausibility, dose–response, and intervention effects, the overall causal inference is moderate for acute noise and ototoxic/ischemic injury (early ROS surges with partial reversal by antioxidants), low-to-moderate for age-related hearing loss (confounding from cumulative exposures and comorbidities), and low for ISSNHL due to mixed clinical results and probable etiologic heterogeneity.

### 3.5. Information Gaps and Priorities for Future Studies

Across included studies, noise paradigms, species/ages, dosing/timing, and outcome measures vary widely, limiting cross-study synthesis and precluding quantitative meta-analysis; harmonized designs and core outcome sets are needed. Many protective effects are demonstrated in animals or small cohorts. Rigorous randomized trials should define optimal timing, dosing, and combinational strategies (e.g., aqueous- plus membrane-phase antioxidants; antioxidant plus Nrf2 activation). Prospective validation of oxidative-stress biomarkers (e.g., 4-HNE, MDA, global oxidant indices) to identify high-risk patients and monitor treatment response remains limited. Genetic and microvascular endophenotypes (e.g., NOX isoform activity, endothelial dysfunction) warrant integration into trial designs. Evidence beyond NIHL, ARHL, and ISSNHL (e.g., Ménière’s disease, autoimmune inner-ear disease) is sparse and should be expanded to define where antioxidant strategies are most impactful.

### 3.6. Shared and Distinct Pathophysiology Across NIHL, ARHL, and ISSNHL

NIHL, ARHL, and ISSNHL share core oxidative mechanisms—early mitochondrial ROS generation, lipid peroxidation (4-HNE, MDA), and downstream apoptotic/necrotic pathways in OHCs and SGNs. However, their temporal dynamics, initiating triggers, and dominant injury compartments differ. NIHL is characterized by an acute oxidative burst within hours of exposure, with prominent OHC and synaptic injury; antioxidant efficacy is greatest when administered pre- or peri-exposure. ARHL reflects cumulative mitochondrial damage, impaired redox buffering, and microvascular/endothelial dysfunction that evolve over years; antioxidant benefits appear modest and likely require long-term, multi-target approaches. ISSNHL presents abruptly and heterogeneously; microvascular compromise, sterile inflammation, and oxidative stress may co-exist, but clinical trials show mixed responses to single-agent radical scavengers, suggesting etiologic subtypes with distinct therapeutic windows. These distinctions rationalize why mechanisms or agents are not interchangeable across etiologies and emphasize the need for stratified trial designs.

### 3.7. A Redox-Centered Conceptual Framework and Classification of Hearing Loss

We propose a framework that organizes hearing loss along six redox-relevant dimensions—trigger and time course, dominant cochlear compartment, ROS source, biomarker kinetics, therapeutic window, and mechanism-matched intervention—thereby linking pathophysiology to testable strategies and explaining variability in antioxidant responses. In NIHL, acute acoustic overexposure produces a rapid mitochondrial/NOX-mediated ROS surge with early increases in 4-HNE and MDA; injury is centered in outer hair cells and ribbon synapses, creating a narrow therapeutic window peri- or very early post-exposure in which fast-acting radical scavengers or rapid Nrf2 activation are most plausible, and intratympanic delivery should be compared with systemic routes using early endpoints such as ABR, pure-tone thresholds, and synaptopathy metrics. In ARHL, cumulative metabolic stress, mitochondrial aging, and microvascular dysfunction generate chronic mitochondrial/NOX-derived ROS with persistently elevated oxidative markers; injury spans outer hair cells, spiral ganglion neurons, and the stria vascularis, supporting a prolonged, preventive window in which multi-target regimens that combine aqueous- and membrane-phase antioxidants with or without Nrf2 activation are rational, with emphasis on long-term outcomes and biomarker-verified target engagement. In ISSNHL, the presentation is abrupt and heterogeneous, with injury compartments and ROS sources varying by subtype (microvascular, inflammatory, idiopathic) and with biomarker timing often uncertain; an early but subtype-dependent window is likely, so trials should stratify patients and test subtype-specific strategies—such as ischemia–reperfusion-oriented antioxidants in suspected microvascular cases or combined antioxidant and anti-inflammatory approaches when inflammation is implicated. Collectively, this framework provides a common language for aligning mechanisms with endpoints and for designing focused, etiology- and timing-specific interventions.

### 3.8. Limitations

This review supports an important role for free radicals and ROS in the pathogenesis of noise-induced, age-related, and idiopathic sudden sensorineural hearing loss; however, several limitations should be considered. First, the included studies varied widely in design, populations, and methods—encompassing differences in noise paradigms, species/ages, dosing and timing of interventions, and outcome measures—potentially limiting generalizability and comparability. Second, many studies were cross-sectional or short-term, providing only a snapshot of ROS involvement; longitudinal studies are needed to clarify temporal dynamics and causal relationships. Third, our review primarily focused on NIHL, ARHL, and ISSNHL; conditions such as Ménière’s disease and autoimmune inner-ear disease were not adequately explored. Fourth, important modifiers—including genetic predisposition, environmental exposures, comorbidities, concomitant therapies, and lifestyle factors—were not uniformly controlled, introducing potential confounding. Fifth, although animal models offer mechanistic insight, translation to clinical practice requires well-designed randomized trials that standardize endpoints and rigorously evaluate efficacy and safety in humans. Finally, because the search was restricted to English-language publications, language and publication bias cannot be excluded. The heterogeneity of available data—across exposures, biomarkers, and outcome measures—also precluded a quantitative meta-analysis and necessitated a qualitative, theme-based synthesis.

## 4. Summary

**Figure 2:**
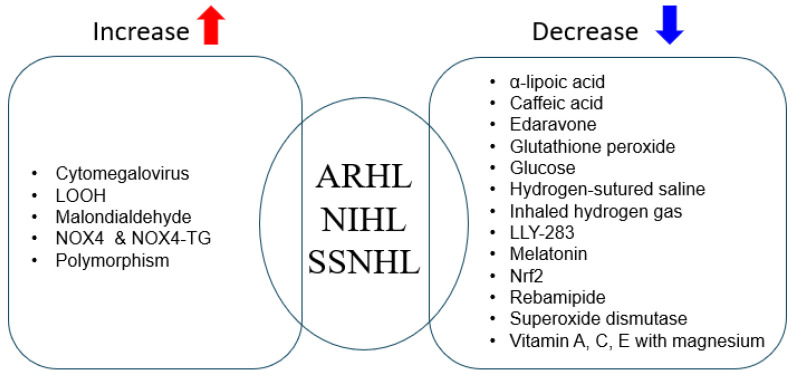
Factors implicated in the onset or exacerbation of hearing loss via ROS, and agents reported to prevent or ameliorate hearing loss through antioxidant or redox-modulating actions. Arrows: the red upward arrow indicates factors that increase ROS levels or hearing-loss risk; the blue downward arrow indicates agents that decrease oxidative injury or mitigate hearing loss.

(1) To elucidate the role of ROS in the pathogenesis of hearing loss, we reviewed 18 studies. Seventeen reported that ROS contribute to the pathogenesis of hearing loss, while one reported no association with the underlying pathophysiology. (2) The primary clinical entities examined were NIHL, ARHL, and ISSNHL. (3) Factors and ROS-related markers associated with the onset or worsening of hearing loss included cytomegalovirus infection, genetic polymorphisms (e.g., NOS3, CAV1), NOX4 expression and NOX-Tg, LOOH, and MDA. Infections or increased expression/levels of these factors were linked to development or aggravation of hearing loss. (4) Antioxidants and redox-modulating interventions evaluated included vitamins A, C, and E with magnesium; rebamipide; α-lipoic acid; LLY-283; edaravone; melatonin; glutathione peroxidase; superoxide dismutase; glucose; hydrogen-saturated saline; activation of Nrf2; inhaled hydrogen gas; and caffeic acid. In most studies, administration or activation of these agents/pathways prevented or ameliorated hearing loss (Figure 2).

## 5. Conclusions

This review supports a contributory role of ROS in noise-induced, age-related, and idiopathic sudden sensorineural hearing loss. Although curative therapies are not available, selected antioxidant/redox-modulating approaches show promise when matched to disease timing and mechanism. To translate these signals into practice, future studies should use etiology- and timing-specific designs—peri-exposure prophylaxis or very-early post-exposure treatment for NIHL; long-term, multi-target regimens (e.g., aqueous- plus membrane-phase antioxidants and/or Nrf2 activation) for ARHL; and stratified ISSNHL trials that distinguish ischemic/microvascular, inflammatory, and idiopathic subtypes. Trials should harmonize exposure paradigms, dosing schedules, and core outcomes (ABR and pure-tone thresholds, speech-in-noise, and patient-reported measures) with predefined effect sizes. Prospective validation of oxidative-stress biomarkers (e.g., 4-HNE, MDA, global oxidant indices) and pharmacodynamic readouts, together with genetic and microvascular endophenotypes, can enable better patient selection and confirm target engagement. Comparative-effectiveness studies are needed to determine when radical scavengers, Nrf2 activators, or anti-inflammatory agents—and which combinations—are most effective. Preregistered protocols, blinded assessments, adequate power, and open data will reduce bias and facilitate future meta-analyses. These focused steps provide a feasible path from mechanistic insight to evidence-based, time-sensitive interventions for hearing-loss prevention and treatment.

## Figures and Tables

**Table 2:** Studies identifying free radicals and ROS as etiologic factors in hearing loss.

Author [Reference]	Study Design	Species and/or Sample	Detection Method	Target Gene(s)	Results/Conclusions
Morioka S, et al., 2018 [16]	Animal study	108 mice	ABR, immunohistochemistry	NOX4	NOX4-Tg mice normally maintained normal hearing, hearing loss at high frequency (12, 20 kHz) is greater after noise exposure. Hsp47 protein increases expression in cochlear and heart of NOX4-Tg mice. NOX4 mice are suitable for ROS hypergenic animal models with auditory sensitivity to noise. Antioxidant therapy can be an effective approach to noise-induced hearing loss. Hsp47 is noted as a potential endogenous defense mechanism against ROS-induced damage.
Kurasawa S, et al., 2023 [17]	Animal study	128 mice	ABR, immunohistochemistry	NOX4	ROS production in NOX4-Tg mice led to a significant decrease in synaptic ribbon components ROS are at least one of the causes of cochlear synaptopathy induced by aging. Thus, protection of synaptic ribbons and reduction in ROS levels are promising approaches to developing novel therapeutic strategies for acquired SNHL.
Teranishi M, et al., 2013 [21]	Case-control genetic association study	2131 patients	DNA, PCR-based genotyping, pure-tone audiometry	NOS3	NOS3 is Significantly associated with increased risk of SSNHL NOS3 and Cav1 gene polymorphisms are genetic risk factors for SSNHL and Ménière’s disease, respectively, suggesting that oxidative stress and vascular dysfunction may play a key role in the pathogenesis of these inner ear disorders.
Capaccio P, et al., 2011 [22]	Human case-control study	109 humans	ROS measurement	ROS	ROS levels were significantly higher in patients with ISSNHL. There was no correlation between ROS and smoking, or between oxidative markers and recovery outcome. The study provides evidence that oxidative stress is significantly associated with ISSNHL. Even with normal antioxidant capacity, an imbalance due to high ROS may contribute to cochlear microvascular damage, suggesting a vascular-origin hypothesis of sudden hearing loss.
Liu C, et al., 2022 [24]	Animal study	100 mice	ABR, immunohistochemistry	PRMT5	Noise exposure could induce hair cell death, cochlear synaptic ribbon loss, and NIHL. Mechanism might involve alleviation of ROS accumulation and activation of the PI3K/AKT pathway, implying that LLY-283 might be a potential candidate for a therapeutic intervention against NIHL.
Kaygusuz I, et al., 2001 [25]	Human study	80 males	Audiological testing	MDA (GSH-Px)	Workders in high noise exposure showed significant hearing loss and significantly elevated MDA and GSH-Px levels. These findings suggest a strong role of free oxygen radicals in noise-induced hearing loss, and that natural antioxidants like GSH-Px are upregulated but insufficient to prevent cochlear damage.
Celik M, et al., 2019 [26]	Prospective controlled human study	50 humans	Audiological assessments	LOOH	No significant correlation was found between oxidative stress markers and IT-MAIS, MUSS, or FFA results. Patients with prelingual profound SNHL are under oxidative stress, and cochlear implantation does not significantly reduce oxidative stress levels within 6 months post-surgery. The authors suggest further long-term studies to clarify the biological mechanisms.
Pecha PP, et al., 2020 [28]	Animal study	40–50 murine	ABR, DPOAE hearing test, dihydroethidium flurorescene staining, immunostaining for cleaved caspase-3, scanning electron microscopy, GFP-tagged CMV	Nrf2	CMV infection significantly increased ROS production in the cochlea and caused hearing loss and outer hair cell damage. Nrf2 knockout mice showed greater hearing loss after CMV infection, indicating the importance of the antioxidant response. in mice, and antioxidant treatment provides partial otoprotection. Antioxidant therapy may be a promising strategy for preventing hearing loss in congenital CMV infection.
Le Prell CG, et al., 2007 [30]	Animal study	29 guinea pigs	ABR	N/A	The combination of vitamins A, C, and E with magnesium (ACEMg) significantly reduced ABR threshold shifts and outer hair cell loss in the cochlea compared to control or single-agent groups. Single treatments (ACE or Mg alone) showed minimal or no statistically significant effect. Suggests synergistic protection from antioxidants and magnesium against noise-induced hearing loss. The findings provide strong rationale for human clinical trials to test similar interventions.
Takumida M, et al., 2009 [31]	Clinical pilot study	46 patients	Pure-tone audiometry	N/A	Hearing thresholds significantly improved at all tested frequencies after 8 weeks of treatment. Better improvements were seen in ears with more severe baseline hearing loss. Treatment with free radical scavengers (rebamipide, α-lipoic acid, vitamin C) significantly improved hearing in elderly patients with ARHL. This therapy shows promising potential as a new clinical approach to treat or possibly prevent age-related sensorineural hearing loss. However, results are preliminary, and longer-term, controlled studies are needed.
Maetani T, et al., 2003 [33]	Animal study	18 gerbils	ABR	N/A	Loss of OHC was not significantly different between each group, and edarabone significantly reduces IHC loss and prevents hearing impairment. Edaravone protects the cochlea against damage caused by transient ischemia, indicating that free radicals play an important role in ischemia-related cochlear injury. Free-radical scavengers may therefore be useful in the treatment of SNHL.
Karlidag T, et al., 2002 [36]	Animal study	50 guinea pigs	Electrophysiological assessment, biochemical analysis	N/A	Melatonin dosing group has less MDA increase, GSH-Px activity is maintained. Melatonin is effective in suppressing noise-induced oxidative damage and preventing hearing loss, and can be a promising therapeutic alternative for the prevention of noise-induced hearing loss.
Xiong H, et al., 2021 [37]	Animal study	100 mice	ABR	N/A	In cochlear explants glucose mitigated H_2_O_2_-induced cytotoxicity, oxidative stress, ATP and NADPH depletion. This study suggests that short-term glucose administration is a simple and effective strategy to prevent oxidative stress-related hearing loss, especially in noise-induced contexts.
Chen L, et al., 2014 [38]	Animal study	55 guinea pigs	ABR, DPOAE	MDA, LP, hydroxyl	The hydrogen-saturated saline group showed significantly less ABR threshold shifts, better-preserved DPOAE amplitudes, and minimal hair cell damage compared to the normal saline group. These findings suggest that hydrogen-saturated saline protects the cochlea from noise-induced hearing loss by reducing oxidative stress.
Honkura V, et al., 2016 [40]	Animal study, human study	50 mice, 602 males	ABR	Nrf2	Mice with loss of Nrf2 showed significantly more hearing and hair cell damage. Nrf2 is a crucial protective factor against NIHL. Pre-activation of Nrf2 can prevent oxidative damage and hearing loss. The human genetic link further supports its potential as a therapeutic target.
Paciello F, et al., 2020 [43]	Animal study	113 rats	ABR	ROS, Nrf2	Rats administered with caffeic acid significantly reduced hearing loss due to noise. Caffeic acid provides otoprotective effects against noise-induced hearing loss. Thus, caffeic acid is a promising natural therapeutic compound for preventing cochlear damage from noise.
Kurioka T, et al., 2014 [45]	Animal study	40 guinea pigs	ABR	ROS	OHC loss in basal and middle cochlear turns was significantly reduced in 1.0% and 1.5% H_2_-treated groups. 1.0% and 1.5% H_2_ groups showed significantly smaller ABR threshold shifts. Inhaled hydrogen gas at concentrations ≥1.0% is effective in reducing ROS production, protecting cochlear hair cells, and preventing NIHL, especially in middle-to-high frequency ranges. The study suggests hydrogen gas as a potential non-toxic therapeutic strategy for acute sensorineural hearing loss.

Abbreviations: ABR, auditory brainstem response; DPOAE, distortion product optoacoustic emissions; GFP, green fluorescent protein; CMV, cytomegalovirus; LP, lipid peroxidation; Nrf2, nuclear factor erythroid 2-related factor 2; ROS, reactive oxygen species; ARHL, age-related hearing loss; PCR, polymerase chain reaction; SSNHL, sudden sensorineural hearing loss; SNHL, sensorineural hearing loss; NIHL; noise-induced hearing loss; OHC, outer hair cell; MDA, malondialdehyde; GSH-Px, glutathione peroxidase; ISSNHL, idiopathic sudden sensorineural hearing loss.

## Data Availability

Not applicable.
